# Impaired Antibody Response Causes Persistence of Prototypic T Cell–Contained Virus

**DOI:** 10.1371/journal.pbio.1000080

**Published:** 2009-04-07

**Authors:** Andreas Bergthaler, Lukas Flatz, Admar Verschoor, Ahmed N Hegazy, Martin Holdener, Katja Fink, Bruno Eschli, Doron Merkler, Rami Sommerstein, Edit Horvath, Marylise Fernandez, André Fitsche, Beatrice M Senn, J. Sjef Verbeek, Bernhard Odermatt, Claire-Anne Siegrist, Daniel D Pinschewer

**Affiliations:** 1 Department of Pathology and Immunology, University of Geneva, Geneva, Switzerland; 2 Institute for Clinical Pathology, University Hospital of Zurich, Zurich, Switzerland; 3 Department of Neuropathology, Georg-August-University Goettingen, Goettingen, Germany; 4 WHO Collaborating Centre for Neonatal Vaccinology, University of Geneva, Geneva, Switzerland; 5 Institute for Clinical Pathology, University Hospital of Zurich, Zurich, Switzerland; 6 Department of Human Genetics, Leiden University Medical Center, Leiden, The Netherlands; 7 Department of Pediatrics, University of Geneva, Geneva, Switzerland; Weatherall Institute of Molecular Medicine, United Kingdom

## Abstract

CD8 T cells are recognized key players in control of persistent virus infections, but increasing evidence suggests that assistance from other immune mediators is also needed. Here, we investigated whether specific antibody responses contribute to control of lymphocytic choriomeningitis virus (LCMV), a prototypic mouse model of systemic persistent infection. Mice expressing transgenic B cell receptors of LCMV-unrelated specificity, and mice unable to produce soluble immunoglobulin M (IgM) exhibited protracted viremia or failed to resolve LCMV. Virus control depended on immunoglobulin class switch, but neither on complement cascades nor on Fc receptor γ chain or Fc γ receptor IIB. Cessation of viremia concurred with the emergence of viral envelope-specific antibodies, rather than with neutralizing serum activity, and even early nonneutralizing IgM impeded viral persistence. This important role for virus-specific antibodies may be similarly underappreciated in other primarily T cell–controlled infections such as HIV and hepatitis C virus, and we suggest this contribution of antibodies be given consideration in future strategies for vaccination and immunotherapy.

## Author Summary

Persistent viruses such as hepatitis C virus (HCV) or HIV can defeat the body's defense system and cause devastating epidemics worldwide. Recent attempts at vaccinating against HIV have relied on the induction of specific antiviral killer T lymphocytes but have failed to confer protection on the host. Better knowledge about how a successful defense should operate is therefore essential for developing and refining new vaccines. Here, we have used a prototypic mouse model to investigate basic defense mechanisms required to eliminate persisting viruses. Experiments in several genetically engineered mouse models show that contrary to common belief, not only antiviral killer T cells, but also antibodies (produced by B cells), are needed to prevent a virus from persisting in its host. These findings suggest that induction of antibodies, along with antiviral killer T lymphocytes, should be envisaged when devising new strategies for vaccinating against HIV or HCV.

## Abstract 3

Andreas Bergthaler and colleagues reveal that T cell control of systemic persistent viral infections requires support by antibodies produced by B cells, which may have important implications for future vaccination strategies against HIV and hepatitis C.

## Introduction

Infections associated with persistent viremia include human immunodeficiency virus (HIV) and the hepatitis B and C viruses (HBV, HCV), which affect more than 500 million people worldwide. However, available options to prevent and treat particularly HIV and HCV are unsatisfactory. To refine existing strategies aimed at combating these devastating epidemics, and to help direct future efforts, a better understanding of the immune effector pathways preventing viral persistence is of particular importance.

For almost a century, lymphocytic choriomeningitis virus (LCMV) infection of mice has served as a primary model to study basic mechanisms of the virus–host relationship in persistent infection [1]. It has led to the discovery of several essential concepts [2], including MHC restriction of T cells, viral mutational escape from CD8 cytotoxic T cells (CTL), CTL dysfunction in persistent infection and MHC linkage of virus control. LCMV neutralizing antibody (nAb) responses typically appear late and remain relatively weak [1]. Accordingly, the key role of CTL in controlling and resolving systemic persistent infections has initially been described for LCMV [3–5] with subsequent extension of the concept to important human pathogens such as HIV and HCV. Declining viremia in HIV coincides with the appearance of antiviral CD8 T cells [6,7], and the concept of CTL-mediated HIV control was further strengthened by the association of “protective” HLA molecules with long-term nonprogression in many so-called “elite controllers” [8]. In addition, experimental depletion of CD8 T cells in simian immunodeficiency virus (SIV)-infected macaques also underlined the importance of CTLs in the control of acute, as well as long-term infection [9–11]. Analogous observations were made in HBV- and HCV-infected monkeys [12,13].

Apart from the virtually undisputed contribution of CTLs, evidence has accumulated to suggest that other mechanisms of immune defense are also needed to contain or resolve systemic persistent virus infection. For instance, “protective” HLA alleles are also found in up to one third of individuals with poor or undetectable immune control of HIV infection [14,15], suggesting that even potent CD8 T cell responses are insufficient for HIV control. Conversely, many “elite controllers” lack any of the known “protective” alleles [15]. Moreover, the recent failure of the CD8 T cell–based Merck “STEP” vaccine trial in human HIV infection has alerted the community and has sparked renewed interest in complementary mechanisms that may aid immune defenses against persistent viral disease [16].

Antibodies are among the obvious candidates to complement CTL-mediated control. However, their contribution to the resolution of primary virus infections in general, and persisting ones in particular, has remained controversial. Rapid mutational escape of persisting viruses from antibody neutralization represents a major obstacle to efficient antibody-mediated control [17–21]. Moreover, observations that patients with Bruton's agammaglobulinemia can control acute viral diseases [22] helped create a generally held notion that, unlike what applies for protection against reinfection, primary viral infections were predominantly controlled by cell-mediated immunity [22]. Experiments in mice, monkeys, and man had shown that passive administration of potent nAbs or transgenic expression of a virus-neutralizing B cell receptor (BCR) can prevent infection [23,24], augment virus control during infection [25–27], or prevent the establishment of persistence [28,29]. Still, these experimental observations did not challenge the above dogma since the experimental conditions chosen did not mimic the kinetics and magnitude of the host's spontaneous nAb response (delayed and weak).

Similarly, it seemed unlikely that antibodies could influence LCMV control and persistence, until B cell–deficient mice were found to control the infection only transiently, or not at all. B cell–deficient mice showed vanishing CD8 T cell function and viral recrudescence [30,31], but the conclusions became doubtful when the mice were shown to have a distorted splenic microarchitecture and intrinsically defective CD4 T cell responses [32–34]. As CD4 T cells are essential to the maintenance of effective antiviral CD8 T cell responses [35], the shortcomings in viral resistance were concluded to result from defective T help, rather than from the lack of antibody [34].

Given the outlined uncertainties, combined with the importance of such fundamental knowledge in order to refine preventive and therapeutic strategies in humans, we have readdressed the role of specific antibody responses to the control and resolution of persistent infection. We used the LCMV model to establish viral infection in genetically engineered mice that support the development of B cells, but do so only with restricted diversity and predominantly LCMV-unrelated specificity. In addition, we infected B cell–sufficient mouse models, unable to mount either serum immunoglobulin M (IgM) or immunoglobulin G (IgG) responses. Our studies reveal that virus-specific antibodies, including early adaptive IgM responses, play an essential role in reducing viral loads and ultimately determine viral clearance or persistence.

## Results

### B Cell Receptor Diversity Correlates with the Magnitude of Antiviral Antibody Responses and Determines Efficacy of Virus Control

**Figure 1:**
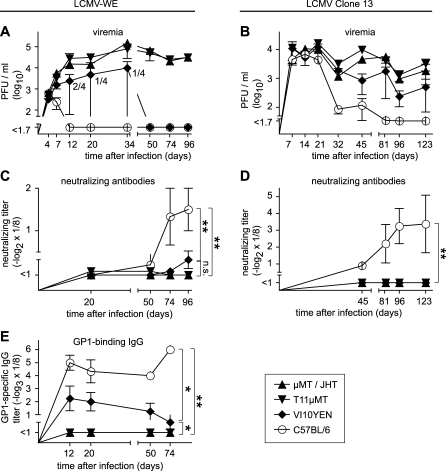
Viral Clearance or Persistence Depends on BCR Repertoire Diversity and Correlates with Antiviral Antibody Formation B cell–deficient (μMT in [A, C, and E]; JHT in [B and D]), BCR-restricted T11μMT and VI10YEN mice, and control C57BL/6 mice were infected i.v. with 10^6^ PFU of LCMV-WE (A, C, and E) or 10^6^ PFU of LCMV Clone 13 (B and D). (A and B) Infectious virus in blood was monitored over time. Numbers next to the VI10YEN curve in (A) indicate viremic VI10YEN mice per number of mice tested at each time point. Comparison of viral clearance kinetics (combined analysis of up to three experiments): LCMV-WE (A) C57BL/6 versus all other groups, VI10YEN versus T11μMT, VI10YEN versus μMT, *p* < 0.01. LCMV Clone 13 (B) C57BL/6 versus all other groups *p* < 0.05. All other comparisons *p* > 0.05. (C and D) nAbs were monitored over time. Double asterisks (**) indicate *p* < 0.01. (E) LCMV-WE-GP1–specific IgG was measured by ELISA. Symbols represent the mean ± SEM of four mice per group. A single asterisk (*) indicates *p* < 0.05, and double asterisks (**) indicate *p* < 0.01. For (A and C), one representative experiment of three similar experiments is shown. (E) displays one of two experiments, with symbols representing the mean ± SEM of three to five mice per group.

Using the murine model of LCMV infection, we aimed here at investigating the contribution of specific antibody to prevent persistent infection. To overcome the limitations intrinsic to B cell–deficient mouse models (i.e., distorted splenic microarchitecture with resulting defects in CD4 T cell responses), we first exploited two genetically engineered mouse models with a severely narrowed, predefined BCR repertoire of LCMV-unrelated specificity. T11μMT [36] carry an immunoglobulin (Ig) heavy chain transgene in an IgM heavy chain–deficient background, whereas VI10YEN [37] combine an Ig light chain transgene with a knockin at the endogenous Ig heavy chain locus. Both constructs render the respective B cells specific for vesicular stomatitis virus (VSV) that is antigenically unrelated to LCMV (for a more detailed description of these strains, including their residual ability of generating antibody repertoire diversity, see Text S1). Unlike B cell–deficient mice, these animals exhibited a normal splenic microarchitecture in immunohistochemistry, and mounted unimpaired CD4^+^ T cell responses against LCMV, as determined by intracellular staining of interferon γ (IFNγ) upon peptide stimulation (Figure S1 and Text S1). We infected B cell–deficient μMT mice [38] (targeted deletion of the IgM transmembrane domain), BCR-restricted T11μMT and VI10YEN mice, and C57BL/6 control mice with 10^6^ plaque-forming units (PFU) of LCMV intravenously (i.v.) (Figure 1). Unlike C57BL/6 mice that resolved viremia within 12 d, T11μMT mice exhibiting the lowest degree of BCR diversity failed to contain the infection and—like B cell–deficient μMT mice—remained viremic throughout the observation period of 96 d (Figure 1A). Similar, albeit less-pronounced, effects were seen in VI10YEN mice displaying a more diverse BCR repertoire than T11μMT mice. Seven of ten VI10YEN mice tested in three independent experiments exhibited protracted viremia as compared to C57BL/6 wild-type mice (Figure 1A and unpublished data). Even more pronounced was the impact of BCR diversity on the control of the more invasive Clone 13 strain of LCMV (Figure 1B). Only C57BL/6 mice succeeded in resolving viremia, whereas BCR-restricted VI10YEN and T11μMT mice, and B cell–deficient JHT [39] mice (targeted deletion of the immunoglobulin J_H_ locus; JHT and μMT mice were used likewise in this study) remained viremic throughout the observation period of 123 d. Thus, BCR diversity was essential for efficient resolution of LCMV infection. Further support for this notion came from experiments in “quasimonoclonal” (QM) mice [40] with a predefined nitrophenyl-specific B cell repertoire owing to knockin of a rearranged immunoglobulin heavy chain gene in combination with an immunoglobulin light chain transgene (Figure S2). Interestingly also, the requirements for BCR diversity became apparently more stringent as the infection was prone to persistence. That is, VI10YEN mice were able to clear LCMV strain WE (LCMV-WE), albeit with some delay, but they failed at resolving chronic infection with LCMV strain Clone 13. The above patterns of virus control or persistence correlated only to a limited extent with the ability of the respective mouse strains to mount a late virus-neutralizing antibody response (Figure 1C and 1D). In LCMV Clone 13 infection, the appearance of neutralizing serum activity around day 45 after infection coincided with viral clearance. In contrast, a clear rise in LCMV-WE-nAb occurred only between 50 and 74 d after infection, i.e., more than 1 mo after viral clearance from the blood. In C57BL/6 mice, this response was consistently measured although the titers varied considerably between individual animals. With further delay and barely above the detection limit of our assays, nAbs were also measured in some VI10YEN mice (Figure 1C, not statistically significant), providing only partial correlation with this mouse strain's ability to control LCMV-WE infection. In contrast, nAbs always remained below detection levels in viremic T11μMT mice. The lack of temporal association, at least in LCMV-WE infection, between the appearance of nAb and clearance, prompted us to study nonneutralizing antibody (non-nAb) responses. The glycoprotein (GP) is the only surface determinant on LCMV particles. It is synthesized as a precursor protein and is posttranslationally cleaved into GP1 and GP2 subunits that remain noncovalently associated [41]. GP1 makes up an outer globular domain, whereas GP2 forms a membrane-anchored stalk [41]. Hence, GP1 is accessible on the infectious virion surface, rendering this antibody specificity of particular interest. Here, we exploited recently developed ELISA techniques [42] for measuring LCMV-WE GP1-specific antibodies. By day 12 after infection, LCMV-WE evoked a GP1-specific IgG response in C57BL/6 mice and at lower titers also in V10YEN mice, but not in T11μMT mice, correlating with virus control (Figure 1E, B cell–deficient μMT mice shown as negative controls). Thus, the timing of the GP1-binding antibody response as well as the differential magnitude in C57BL/6, VI10YEN and T11μMT mice matched best the pattern of virus clearance.

### Both Adaptive IgM and IgG Are Needed for Efficient Virus Control

**Figure 2:**
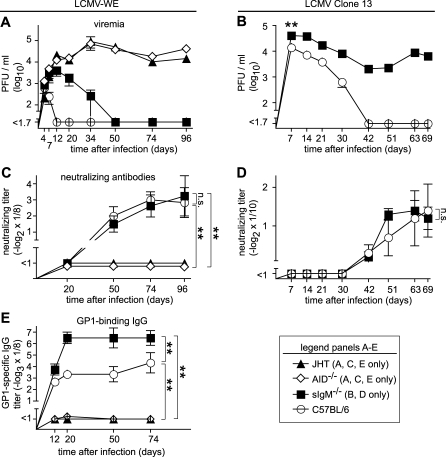
Virus Control Requires Secreted IgM and Also AID-Dependent Class-Switch Recombination and Affinity Maturation (A–E) Mice of the indicated genotypes were infected with 10^6^ PFU of LCMV-WE (A, C, and E) or Clone 13 (B and D) i.v. Viremia (A and B), nAbs (C and D), and LCMV-WE-GP1–binding antibodies (E) were monitored over time. Symbols represent the mean ± SEM of three to four (A, C, and E) or nine to ten (B and D) mice per group. Comparison of viral clearance kinetics: LCMV-WE (A): C57BL/6 versus all other groups, sIgM^−/−^ versus AID^−/−^, sIgM^−/−^ versus JHT, *p* < 0.05. LCMV Clone 13 (B): C57BL/6 versus sIgM^−/−^ *p* < 0.01, as indicated by double asterisks (**). All other comparisons *p* > 0.05. n.s., not significant.

Next, we assessed the individual contribution of IgM and IgG responses to virus control. All monoclonal LCMV nAbs characterized today are of an IgG isotype, and so is the late nAb response observed in the course of natural infection [27]. Hence, any potential role of antibodies in resolution of LCMV infection had previously been accredited to IgG. To test for the role of class switch-dependent isotypes including IgG, we used gene-targeted mice lacking activation-induced cytidine deaminase (AID^−/−^) [43]. AID^−/−^ mice are unable to undergo class-switch recombination and somatic hypermutation, and in our experiments, could not resolve LCMV-WE infection during the observation period of 96 d (Figure 2A). As expected, AID^−/−^ mice displayed a complete absence of nAbs and GP1-specific serum IgG (Figure 2C and 2E), suggesting that immunoglobulin class-switch recombination and IgG production together with somatic hypermutation are essential steps in the resolution of LCMV infection.

To assess a potential role of IgM antibodies we exploited the sIgM^−/−^ mouse model [44]. sIgM^−/−^ B cells express IgM as their surface receptor and secrete IgG upon class-switch recombination but are unable to secrete the early IgM isotype. Experiments were carried out to confirm that according to expectations and unlike B cell–deficient μMT mice, B cell–competent sIgM^−/−^ mice display a normal lymphoid microarchitecture and mount unimpaired CD4^+^ T cell responses (Figure S3). Surprisingly, however, LCMV-WE infection resulted in substantially prolonged viremia in sIgM^−/−^ mice as compared to C57BL/6 control mice (Figure 2A), suggesting that contrary to expectations, an antibody response of IgM isotype contributed to virus control. More strikingly even, nine of ten sIgM^−/−^ mice failed to resolve LCMV Clone 13 infection for a period of at least 100 d, whereas all nine C57BL/6 mice had cleared viremia within 42 d after infection (Figure 2B and unpublished data).

**Figure 3:**
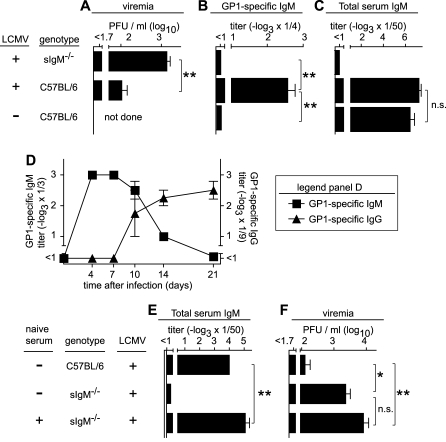
Early Adaptive IgM Response Rather Than Natural Antibodies Contributes to Virus Control (A–C) sIgM^−/−^ and C57BL/6 control mice were infected with 10^6^ PFU of LCMV-WE i.v. Viremia (A), LCMV-WE-GP1–specific IgM (B), and total serum IgM (C) were determined 8 d later. Bars represent the mean ± SEM of five to ten mice per group. (D) C57BL/6 mice were infected with 10^6^ PFU of LCMV-WE i.v. LCMV-WE-GP1–specific IgM and IgG were measured over time. Symbols represent the mean ± SEM of four mice per group. (E and F) sIgM^−/−^ mice were substituted i.p. with 2 ml of naive C57BL/6 serum on day −1. Control groups consisted of sIgM^−/−^ and C57BL/6 mice without serum substitution. All mice were infected with 10^6^ PFU of LCMV-WE i.v. on day 0. Total serum IgM was measured on the day of virus challenge (E), and viremia was determined on day 21 (F). Bars represent the mean ± SEM of five to twelve animals. A single asterisk (*) indicates *p* < 0.05, and double asterisks (**) indicate *p* < 0.01. n.s., not significant.

The analysis of nAb responses (measuring both IgM and IgG, Figure 2C and 2D), confirmed that the kinetics and magnitude of the nAb response were indistinguishable in sIgM^−/−^ and C57BL/6 controls, and therefore, likely were of IgG isotype as previously reported. As expected, also LCMV-WE-GP1–binding IgG responses showed normal kinetics in sIgM^−/−^ mice. Somewhat higher GP1-specific IgG peak titers in sIgM^−/−^ mice as compared to C57BL/6 control mice were likely the result of prolonged viremia with an increased antigen burden (Figure 2E). In support of this notion, differences in antibody titers became particularly apparent between day 12 and 20 when C57BL/6, but not sIgM^−/−^, mice had cleared the infection. The above experiments had suggested that differences in virus loads of sIgM^−/−^ and C57BL/6 mice were manifest as early as 1 wk after infection (*p* < 0.01 for LCMV Clone 13, Figure 2B). Additional experiments corroborated this difference in early virus loads also for LCMV-WE infection (Figure 3A, *p* < 0.01). As a likely mediator of this difference, ELISA assays detected GP1-specific IgM responses in day 8 LCMV-WE–infected C57BL/6, but not sIgM^−/−^, mice (Figure 3B), antibodies that were absent from naive C57BL/6 mouse serum (Figure 3B). Importantly also, the GP1-specific IgM responses measured here were confirmed to be entirely antigen-specific since total serum IgM (Figure 3C), unlike serum IgG [45], remained largely unaltered after LCMV infection. A time-course analysis revealed that GP1-specific IgM was highest on day 4 and 7 after infection, followed by a continuous decline of this isotype concomitant with class switch and appearance of GP1-specific IgG (Figure 3D). These assays were, however, performed with unseparated serum, and competition between IgG and IgM in ELISA may have resulted in an underestimation of IgM levels at later time points.

sIgM^−/−^ mice not only lack adaptive IgM responses but also natural IgM, which contributes to control of other viral infections [46]. For dissecting the role of preexisting natural antibodies, we reconstituted sIgM^−/−^ mice with naive C57BL/6 mouse serum (Figure 3E). Despite reaching total serum IgM levels at least equivalent to normal C57BL/6 mice, reconstitution of natural IgM in sIgM^−/−^ mice failed to restore virus control (Figure 3F). Taken together, these data demonstrated that adaptive IgM as well as IgG responses both played essential roles in the efficient resolution of LCMV infection. Interestingly, unaltered nAb kinetics in sIgM^−/−^ and C57BL/6 control mice suggested that antiviral IgM mediated its effects by mechanisms other than classical virus neutralization.

### Antibody Therapy Prevents Viral Persistence and Preserves CTL Function in T11μMT Mice

**Figure 4:**
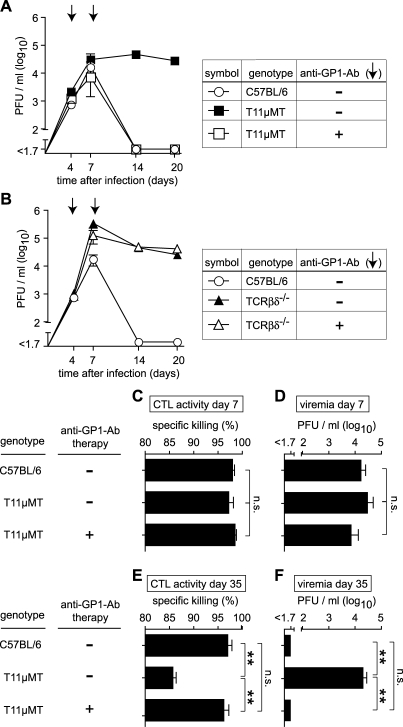
Antibody Therapy Prevents Viral Persistence and Preserves CTL Function in T11μMT Mice (A and B) Mice of the indicated genotypes were infected with 10^6^ PFU of LCMV WE. On day 4 and day 7 (arrows), they were treated with GP1-specific antibody in normal serum or with normal serum only (control), as outlined in the chart. Viremia was monitored over time. Symbols represent the mean ± SEM of four to five mice per group. The data in (A and B) originate from the same experiment, and the identical C57BL/6 group is shown in both graphs. Virus clearance kinetics in C57BL/6 mice and anti-GP1 antibody-treated T11μMT mice were indistinguishable (*p* > 0.05), but each one of them differed significantly from the remaining three groups (*p* < 0.01). All other comparisons *p* > 0.05. (C–F) C57BL/6 and T11μMT mice were treated as in (A and B). On day 7 (C and D) and day 35 (E and F) after infection, virus-specific cytotoxic activity of CD8^+^ T cells was measured in an in vivo CTL assay (C and E), and virus loads were determined in blood (D and F). Bars represent the mean ± SEM of three to five mice per group. Double asterisks (**) indicate *p* < 0.01. n.s., not significant.

Next, we studied whether antibody therapy could restore virus control in BCR-restricted LCMV noncontroller mice. For this purpose, we infected T11μMT mice with LCMV-WE and treated them on day 4 and day 7 with either normal serum (negative control) or with normal serum reconstituted with GP1-specific monoclonal antibody (Figure 4A). T11μMT mice treated with GP1-specific antibody eliminated LCMV as efficiently as did C57BL/6 wild-type mice, whereas control-treated T11μMT mice remained viremic, as expected (compare Figure 1A). The same antibody treatment that was successful in T11μMT mice failed, however, to exert a detectable effect on virus loads when administered to TCRβ^−/−^δ^−/−^ mice [47] lacking T cells owing to homozygous deletion of the T cell receptor β and δ chain loci (Figure 4B). These data were compatible with the interpretation that T cells of T11μMT mice could control LCMV infection if appropriately aided by specific antibodies, whereas neither T cells nor antibody therapy was sufficient to control LCMV on its own.

Exhaustion of CD8 T cell responses as a result of continued antigen exposure is a common observation in persistent viral infection [48,49]. Hence, we investigated whether antibody therapy could prevent CD8 T cell exhaustion in T11μMT mice. The initial LCMV-specific CD8 T cell response of T11μMT mice not only was of normal frequency and was functional in terms of IFNγ secretion (Figure S1D) but also displayed an unimpaired capacity for killing antigenic cells in vivo, irrespective of antibody treatment (Figure 4C). Of note, virus loads were still similar in all groups when these tests were performed on day 7 (Figure 4D). On the contrary, defective cytolytic activity was observed in control-treated T11μMT mice on day 35 during the chronic phase of infection. Prevention of viral persistence by antibody therapy (Figure 4F) restored in vivo cytotoxicity of T11μMT mice to normal levels on day 35 (Figure 4E). This lent further support to the interpretation that the CD8 T cell response of T11μMT mice was intrinsically normal, and that its decline during chronic infection was merely the result of viral persistence rather than the cause thereof. Albeit less likely, a subtle intrinsic CD8 T cell deficiency of T11μMT mice cannot, however, be formally excluded. Irrespective thereof, antibody therapy may help preserve the antiviral CD8 T cell response.

### Unimpaired Virus Clearance in the Absence of the Classical and Alternative Complement Cascades, of the Fc Receptor γ Chain or of Fc γ Receptor IIB

**Figure 5:**
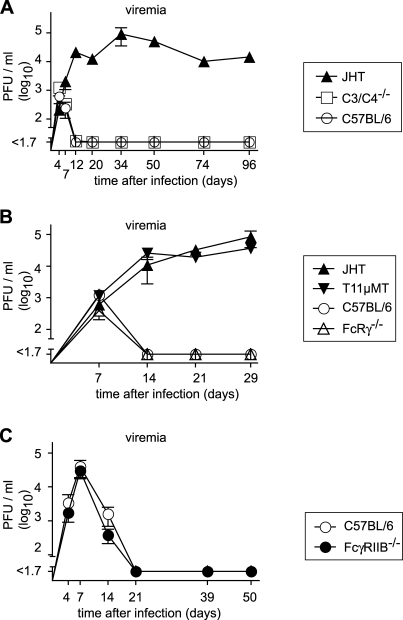
Efficient Virus Clearance Occurs in the Absence of Either Complement, Fc Receptor γ Chain, or FcγRIIB Mice of the indicated genotypes were infected with 10^6^ PFU of LCMV-WE, and viremia was monitored over time. Symbols represent the mean ± SEM of three to four mice per group. (A–C) represent independent experiments. C3^−/−^C4^−/−^ mice were tested alongside the experimental groups displayed in Figure 2A, 2B, and 2C, and hence the identical C57BL/6 and JHT control groups are shown in Figure 2A and (A). Comparison of viral clearance kinetics: (A) C57BL/6 versus C3^−/−^C4^−/−^ *p* > 0.05 (not significant); C57BL/6 versus JHT, C3^−/−^C4^−/−^ versus JHT *p* < 0.05. (B) C57BL/6 versus FcRγ ^−/−^, JHT versus T11μMT *p* > 0.05 (not significant); C57BL/6 versus JHT, C57BL/6 versus T11μMT, FcRγ^−/−^ versus T11μMT, FcRγ^−/−^ versus JHT, *p* < 0.05. (C) C57BL/6 versus FcγRIIB^−/−^ *p* > 0.05 (not significant).

To evaluate the role of the classical and alternative complement cascades as major effector pathways of antibody-mediated immunity, we studied clearance of LCMV-WE in mice lacking complement components C3 and C4 (C3^−/−^C4^−/−^ mice; see Materials and Methods). C3^−/−^C4^−/−^ mice resolved viremia as efficiently as wild-type control mice (Figure 5A), whereas B cell–deficient JHT control mice remained viremic throughout. An analogous experiment was carried out in mice lacking Fc γ receptors I, III, and IV owing to deletion of the common γ chain (FcRγ^−/−^ [50]). FcRγ^−/−^ mice cleared LCMV-WE infection as efficiently as did C57BL/6 wild-type mice, whereas B cell–deficient JHT and T11μMT mice both showed unchecked viremia throughout the observation period (Figure 5B). Unlike Fc γ receptors I, III, and IV, Fc γ receptor IIB (FcγRIIB) expression does not depend on the common γ chain. To study the contribution of this receptor, we infected FcγRIIB -deficient mice (FcγRIIB^−/−^; see Materials and Methods) with LCMV, but found unimpaired virus control (Figure 5C). Taken together, these data excluded an essential individual contribution of classical and alternative complement cascades, of Fc γ receptors I, III, and IV, and of FcγRIIB, respectively, in mediating protective antibody effects in the natural course of LCMV infection.

## Discussion

The present data show that virus-specific antibody responses, including early IgM, play an unexpected key role in preventing viral chronicity in the CTL-controlled murine model of LCMV infection. These observations are compatible with the rapid escape from antibody recognition seen in other primarily CTL-controlled infections, including HIV and HCV [19–21], and indicate that specific antibody responses represent a level of antiviral pressure that tends to be underappreciated.

The observed antiviral effects can only be partially accredited to antibody-mediated virus neutralization. Albeit clearance of LCMV Clone 13 in C57BL/6 mice did coincide with the appearance of nAbs (compare Figure 2B and 2D), IgM effects on LCMV Clone 13 and LCMV-WE titers were evident already on day 7/8 after infection (Figures 2A, 2B, and 3A) at a time when nAbs were undetectable even if using virtually undiluted serum for the assays (unpublished data). Similarly, LCMV-WE was cleared weeks before nAbs could be detected (compare Figure 2A and 2C). Obviously, “absence of proof” for early nAb does not equate “proof of absence,” and we recognize that “nAb consumption” during viremia or subsequent phases of protracted clearance from tissues [51] would provide an explanation for our inability to detect nAbs. However, we favor the idea that the delay in LCMV nAb detection, relative to the antiviral effects observed, rather results from the need for time-intensive affinity maturation [42].

The protective capacity of nAbs is classically explained by “virion occupancy,” i.e., sterical hindrance interfering with cell-surface receptor binding [52]. Non-nAbs, on the other hand, may mediate protection via a number of mechanisms, including: (1) virion occupancy by complement C1q binding to a virion-bound antibody [53], (2) complement cascade activation, leading to further virion occupancy through covalent opsonization, (3) complement-mediated virion lysis, (4) Fc-receptor–mediated virion phagocytosis and destruction, (5) Fc-receptor–mediated stimulation of the innate immune system, (6) immune complex formation and resulting modification of tissue distribution and cellular tropism, (7) antibody-dependent cellular cytotoxicity (ADCC), via antibody binding to viral surface proteins on infected cells, (8) impaired virus production, through antibody-mediated cross-linking of cell surface–expressed viral envelope protein [54], or (9) destruction of target protein or host cells, through antibody-mediated reactive oxygen catalysis [55]. Although a full assessment of the individual contribution of each of these potential pathways lies outside the scope and intention of the present study, we do present data ruling out a major individual contribution for covalent complement opsonization and lysis, mediated through C3 and/or C4 activation, as well as for FcRγ- and FcγRIIB-facilitated mechanisms [50,56] (Figure 5). It remains possible that another mechanism not yet experimentally addressed here may account for most of the antibody effects observed, e.g., Fc α/μ receptor–mediated clearance [57] could explain the observed IgM effects (Figures 2A, 2B, and 3A). However, substantial redundancy in these multiple mechanisms may render it difficult to work out the contribution of individual mechanisms including the ones we have tested here, i.e., in the absence of a specific pathway, compensation by the remaining ones may suffice for virus clearance.

The present findings are of considerable importance for our understanding of virus–host relationship in persistent infection and for refining preventive and therapeutic strategies: The success of antibody therapy in T11μMT mice, but not in TCRβ^−/−^δ^−/−^ animals (Figure 4A and 4B), suggests a synergistic effect of cellular and humoral immune defense, at least for LCMV. Antibody therapy can apparently help preserve T cell function, and hence early administration may be most promising. Albeit our experimental therapy was administered during a phase of infection in which IgM predominates (compare Figure 3D), IgG was efficient. This may be of practical importance since both vaccination and immunotherapy typically rely on IgG rather than on IgM.

Owing to structural reasons, potent nAb responses against persisting viruses are generally difficult to elicit through vaccination [20,58], but non-nAbs represent an attainable goal. The present data from LCMV infection in mice strongly suggest that non-nAbs, alongside antiviral CTL responses and nAbs, can determine clearance or persistence. We suggest that non-nAbs operate by blunting the infection and thereby strengthening the efficacy of other immune mediators such as CTLs and nAbs, but also NK cells [59,60]. In the context of the cited literature, our data support the idea that antibodies should be considered anew in vaccination strategies aimed at combating persistent viral disease, and that aside from nAbs as a vaccine goal, non-nAbs also should be induced and assessed.

It has recently been shown that non-nAbs specific for LCMV GP-1 can mediate protective effects when expressed in a transgenic context [27]. In nontransgenic wild-type mice, we now show that virus-specific antibody responses, including GP-1–binding IgM, are not only generated rapidly (see also Figure 2G), but also exert significant antiviral pressure (compare Figure 2B and 2F; *p* < 0.01) in the days before nAbs become detectable. Although we focused in our assays on GP-1 binding antibodies, it remains entirely possible that additional non-nAbs of alternative specificities may also contribute to the observed protective effects. Defining characteristics and specificities of “protective” and “nonprotective” non-nAbs may therefore represent an important next step in the direction of exploiting the protective capacity of non-nAbs for vaccination and immunotherapy.

Failure of the HIV AIDSVax trial eliciting mostly non-nAbs in the absence of cell-mediated immunity has somewhat dampened the hope that non-nAbs could help containing persistent infections [61,62]. Albeit non-nAbs are apparently unable to protect on their own, studies have correlated ADCC with HIV nonprogression, suggesting that non-nAbs may indeed contribute to long-term control of HIV [63]. However, much remains unclear about the overall importance of non-nAbs, and antibodies in general, to the natural course of HIV infection [64]. Moreover, the available data emphasize that the mechanisms of antibody-mediated protection do not always follow the traditional way of thinking. For instance, even a broadly HIV-neutralizing monoclonal antibody was shown to protect primarily via Fc-receptor–dependent mechanisms [65]. Of note in this context, the HIV envelope displays defective glycoproteins in great abundance [66]. Albeit unable to mediate cell entry, such defective glycoproteins are highly immunogenic and may represent efficient targets for non-nAbs. Of further importance here, non-nAbs have a relatively broad spectrum of activity against both autologous and heterologous HIV strains [67].

Taken together, the results from this study show that CD8 T cells, even if firmly established as the predominant mechanism of antiviral immune defense, need support from specific antibodies to prevail and prevent viral persistence. Given the relative ease of induction of non-nAbs (relative to nAbs), combined with the observed protective effects, our findings may provide new impetus for inclusion of antibody targets in vaccines against persistent viral diseases.

## Materials and Methods

### Mice and animal experiments.

C57BL/6 wild-type mice, μMT^−/−^ [38], JHT^−/−^ [39], T11μMT [36], VI10YEN [37], QM [40], AID^−/−^ [43], sIgM^−/−^ [44], C3^−/−^C4^−/−^ double-deficient mice (a crossbreed of C3^−/−^ [68] and C4^−/−^ [69] mice), TCRβ^−/−^δ^−/−^ [47], and FcRγ^−/−^ [50] were bred at the Institute of Laboratory Animal Science, University of Zurich, and were housed under specific pathogen-free (SPF) conditions throughout. FcγRIIB^−/−^ mice on a pure C57BL/6 background, in which exons 4 and 5, encoding the ligand-binding EC2 and transmembrane (TM) region, have been deleted by gene targeting in Bruce4 ES cells (C57BL/6 background), were generated in the laboratory of Sjef Verbeek. Absence of functional FcγRIIB was confirmed both in functional in vivo and in vitro assays and at the protein level, as will be described elsewhere in more detail. Experiments with FcγRIIB^−/−^ mice and controls were performed in a conventional mouse facility. Animal experiments were carried out at the University of Geneva and the University of Zurich with authorization by the respective cantonal authorities and in accordance with the Swiss law for animal protection.

### Viruses, infection, and antibody therapy.

LCMV-WE was originally obtained from F. Lehmann-Grube (Heinrich-Pette Institut, Hamburg, Germany) and was propagated on L929 cells. LCMV Clone 13 was obtained originally from R. Ahmed (Emory University, Atlanta, Georgia, United States) and was grown on BHK-21 cells. Infections were performed at a standard dose of 10^6^ PFU by the intravenous route. For therapy of T11μMT and TCRβ^−/−^δ^−/−^ mice, GP1-specific monoclonal antibody KL25 [70] was administered intraperitoneally on day 4 (100 μg) and on day 7 (1 mg), reconstituted in 400 μl of normal (nonimmunized and uninfected) C57BL/6 serum. Control animals were given 400 μl of normal serum.

### Virus titration and detection of LCMV-neutralizing antibodies.

LCMV virus stocks and blood samples were titrated by standard immunofocus assays on MC57G cells [71]. nAbs against LCMV-WE and LCMV Clone13 were measured in an immunofocus reduction assay using the respective homologous virus as described [58].

### Enzyme-linked immunosorbent assays (ELISA).

GP1-specific IgM and IgG responses were measured by ELISA using a GP1-Fc fusion construct produced in an eukaryotic system as described [42]. In the GP1-Fc construct, amino acids 1–265 (i.e., the GP1 domain [42]) of the LCMV-WE glycoprotein gene are fused to human Fc. As sole modification to the published method, anti-mouse IgM monoclonal antibody coupled to HRP (Sigma) was used instead of anti-mouse IgG when detecting GP1-specific IgM. Total serum IgM titers were measured in ELISA as described previously [45]. Titers displayed represent the serum dilution yielding twice background optical density values.

### Enumeration of epitope-specific T cell populations and in vivo CTL assay.

Single-cell suspensions of splenocytes were used for intracellular cytokine assays as described [72]. Restimulation of virus-specific cells was performed for 5–6 h in the presence of the following synthetic peptides at 10^−6^ M concentration: KAVYNFATC (GP33, CD8^+^ T cells), GPDIYKGVYQFKSVEFD (GP64, CD4^+^ T cells), and SGEGWPYIACRTSVVGRAWE (NP309, CD4^+^ T cells).

Cytotoxic activity of CD8^+^ T cells was measured in an in vivo CTL assay as previously described [73]. In brief, syngeneic C57BL/6 splenocytes were labeled with the fluorescent dye carboxyfluorescein diacetate succinimidyl ester (CFSE) at two different concentrations (CFSE^high^ or CFSE^low^). In addition, CFSE^high^ cells were pulsed with GP33 peptide at 10^−6^ M concentration for recognition by antiviral CTLs. 3 × 10^7^ cells of each population were cotransferred into virus-infected recipient mice and into naive C57BL/6 mice (control). Five hours later, the percentage of CFSE^high^ and CFSE^low^ donor cells in peripheral blood mononuclear cells was determined by flow cytometry. Specific killing was calculated as: 100 − ([(% CFSE^high^ in test animal / % CFSE^low^ in test animal) / (% CFSE^high^ in naive / % CFSE^low^ in naive)] × 100).

### Immunohistochemistry.

Histological analyses were performed on snap-frozen tissue. Sections were stained with rat monoclonal antibodies against murine B220 (Pharmingen), F4/80, MOMA1, and ERTR9 (all from BMA Biomedicals). Bound antibody was detected using a goat anti-rat antibody (Caltag Laboratories) and an alkaline phosphatase–coupled donkey anti-goat antibody (Jackson ImmunoResearch Laboratories) with naphthol AS-BI (6-bromo-2-hydroxy-3-naphtholic acid 2-methoxy anilide) phosphate and new fuchsin as a substrate. The sections were counterstained with hemalum. For tissues of VI10YEN mice carrying a light-chain transgene with rat constant domains, reaction of anti-rat monoclonal antibody with the transgenic light chain was prevented by using an alkaline phosphatase conjugated Fc γ fragment–specific goat anti-rat IgG antibody as a secondary antibody (Jackson ImmunoResearch Laboratories).

### Statistical analysis.

One-way analysis of variance (ANOVA) with the Least Significant Difference (LSD) post test was used for the comparison of individual values from multiple groups. Two-way ANOVA was performed to compare antibody responses over time. ANOVA was performed with SPSS version 13.0. Differences in individual values between two groups were analyzed by *t-*tests (unpaired, two-tailed), and virus clearance kinetics were compared in log-rank tests using GraphPad Prism software vs. 4.0b. Viral titers were log-transformed for statistical analysis, and viral clearance kinetics were compared in a Kaplan-Meier format. *p*-Values < 0.05 were considered statistically significant; *p*-values < 0.01 were considered highly significant.

## Supporting Information

Figure S1 Normal Splenic Microarchitecture and Unimpaired CD4^+^ and CD8^+^ T Cell Responses in T11μMT and VI10YEN Mice (A) Histological spleen sections of μMT, VI10YEN, T11μMT, and C57BL/6 control mice were stained for B220 (B cells), F4/80 (red pulp macrophages), ERTR9 (marginal zone macrophages), or MOMA-1 (metallophilic marginal zone macrophages) as indicated. Each image displays a representative area of spleen from three age-matched mice per group analyzed. Magnification bars indicate 200 μm.
(B): Mice of the indicated genotypes were infected with 10^6^ PFU of LCMV-WE i.v. Eight days later, epitope-specific CD4^+^ (GP64 and NP309) and CD8^+^ (GP33) T cell frequencies in spleen were determined in an intracellular cytokine assay. Bars represent the mean ± the standard error of the mean (SEM) of three (GP64, NP309) or three to eight animals (GP33) per group.
(4.6 MB TIF)

Figure S2 Delayed Virus Clearance in Quasimonoclonal (QM) Mice Mice of the indicated genotypes were infected with 10^6^ PFU of LCMV-WE. Viremia (A) and nAbs (B) were monitored over time. Symbols represent the mean ± SEM of two to five mice per group. One representative experiment of two similar ones is shown. Comparison of viral clearance kinetics (combined analysis of two experiments): QM versus C57BL/6, QM versus T11μMT, C57BL/6 versus T11μMT *p* < 0.01.
(453 KB EPS)

Figure S3 Normal Splenic Microarchitecture and Unimpaired CD4^+^ and CD8^+^ T Cell Responses in sIgM^−/−^ Mice (A) Histological spleen sections of sIgM^−/−^ were stained for B220 (B cells), F4/80 (red pulp macrophages), ERTR9 (marginal zone macrophages), or MOMA-1 (metallophilic marginal zone macrophages) as indicated. Sections of μMT and C57BL/6 mice are shown for comparison (same panels as in Figure S1A). Each image displays a representative area of spleen from three age-matched mice per group analyzed. Magnification bars indicate 200 μm.
(B) sIgM^−/−^ and C57BL/6 control mice were infected with 10^6^ PFU of LCMV-WE i.v. Eight days later, epitope-specific CD4^+^ (GP64 and NP309) and CD8^+^ (GP33) T cell frequencies were determined in an intracellular cytokine assay. Bars represent the mean ± SEM of three mice per group.
(5.9 MB TIF)

Text S1 Normal Splenic Microarchitecture and Unimpaired CD4+ T Cell Responses in Mice with Restricted B Cell Receptor Diversity (65 KB DOC)

## Abbreviations

AID: activation-induced cytidine deaminase
BCR: B cell receptor
CTL: cytotoxic T lymphocyte
GP: glycoprotein
HCV: hepatitis C virus
HIV: human immunodeficiency virus
IgM: immunoglobulin M
LCMV: lymphocytic choriomeningitis virus
nAb: neutralizing antibody
non-nAb: nonneutralizing antibody
PFU: plaque-forming unit
QM: quasimonoclonal
