# Supplementary figures and images for: Impaired Antibody Response Causes Persistence of Prototypic T Cell–Contained Virus
Source: PLoS Biol. 2009 Apr 7;7(4):e1000080. doi: 10.1371/journal.pbio.1000080 (PMC2672599; doi:10.1371/journal.pbio.1000080)

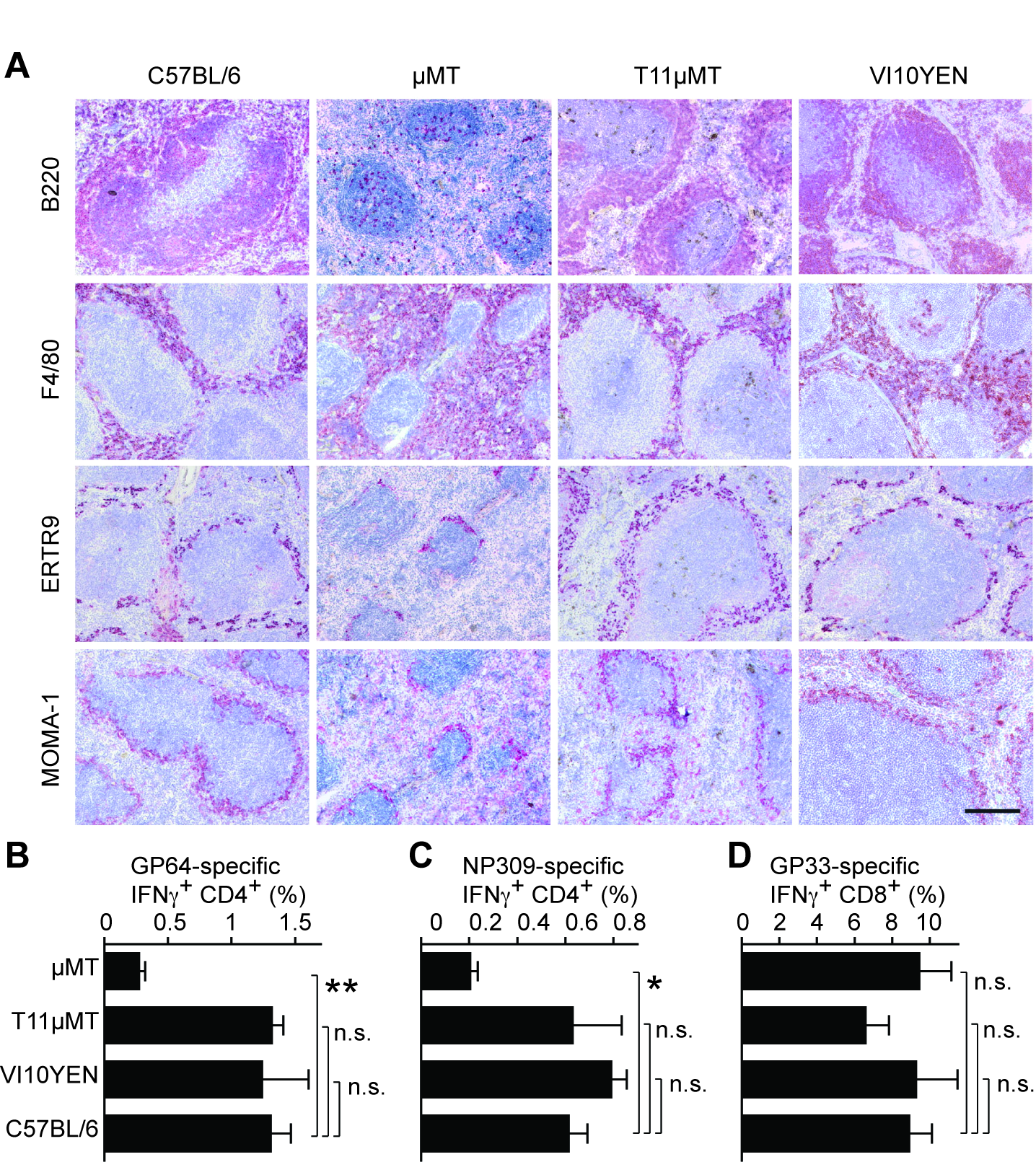

Supplement: Figure S1 — (A) Histological spleen sections of μMT, VI10YEN, T11μMT, and C57BL/6 control mice were stained for B220 (B cells), F4/80 (red pulp macrophages), ERTR9 (marginal zone macrophages), or MOMA-1 (metallophilic marginal zone macrophages) as indicated. Each image displays a representative area of spleen from three age-matched mice per group analyzed. Magnification bars indicate 200 μm. (B): Mice of the indicated genotypes were infected with 106 PFU of LCMV-WE i.v. Eight days later, epitope-specific CD4+ (GP64 and NP309) and CD8+ (GP33) T cell frequencies in spleen were determined in an intracellular cytokine assay. Bars represent the mean ± the standard error of the mean (SEM) of three (GP64, NP309) or three to eight animals (GP33) per group. (4.6 MB TIF) [file pbio.1000080.sg001.tif]

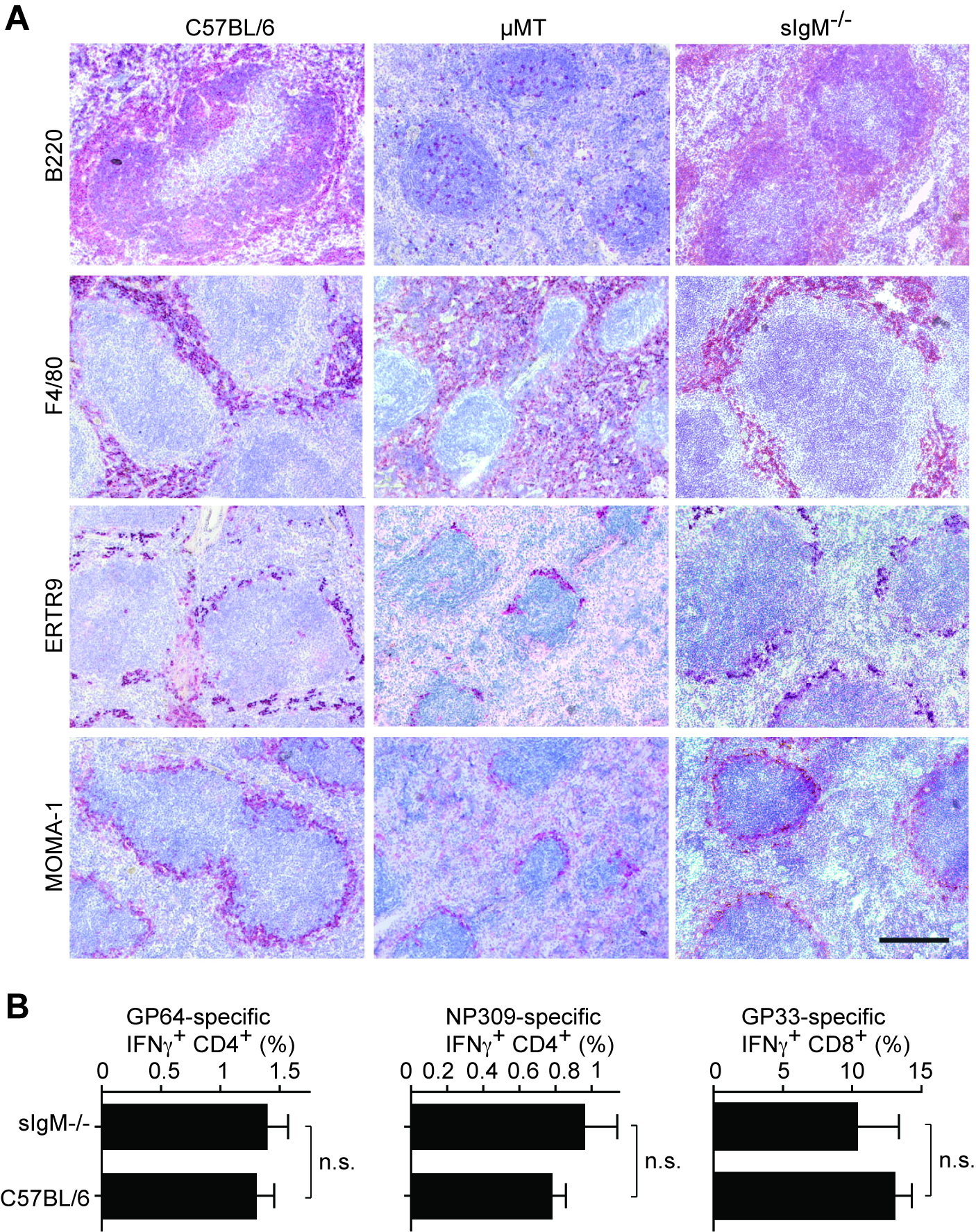

Supplement: Figure S3 — (A) Histological spleen sections of sIgM−/− were stained for B220 (B cells), F4/80 (red pulp macrophages), ERTR9 (marginal zone macrophages), or MOMA-1 (metallophilic marginal zone macrophages) as indicated. Sections of μMT and C57BL/6 mice are shown for comparison (same panels as in Figure S1A). Each image displays a representative area of spleen from three age-matched mice per group analyzed. Magnification bars indicate 200 μm. (B) sIgM−/− and C57BL/6 control mice were infected with 106 PFU of LCMV-WE i.v. Eight days later, epitope-specific CD4+ (GP64 and NP309) and CD8+ (GP33) T cell frequencies were determined in an intracellular cytokine assay. Bars represent the mean ± SEM of three mice per group. (5.9 MB TIF) [file pbio.1000080.sg003.tif]
