# Supplementary material for: Impaired Antibody Response Causes Persistence of Prototypic T Cell–Contained Virus
Source: PLoS Biol. 2009 Apr 7;7(4):e1000080. doi: 10.1371/journal.pbio.1000080 (PMC2672599; doi:10.1371/journal.pbio.1000080)
Supplement: Text S1 — (65 KB DOC) [file pbio.1000080.sd001.doc]

Supporting Text S1

**Normal splenic microarchitecture and unimpaired CD4^+^ T cell responses in mice with restricted B cell receptor diversity**

T11µMT mice [36] carry a immunoglobulin heavy chain (IgH) transgene (T11) derived from a monoclonal nAb against vesicular stomatitis virus (VSV). This transgene is expressed on a Ig µ-chain-deficient (µMT [38]) background, and hence the IgH chain is exclusively transgene-derived and of the IgM isotype. Residual BCR diversity can only be generated by at random pairing with endogenous BCR light chains. Neither affinity maturation nor isotype class switch can occur at the transgenic IgH locus which is randomly inserted in the mouse genome. VSV is antigenically unrelated to LCMV, the model infection studied here, and thus there is no recognition of LCMV antigens by VSV-specific T11µMT B cells. VI10YEN mice [37] carry a BCR IgH locus knock-in (heterozygous) plus a BCR light chain (IgL) transgene, both of them also derived from a monoclonal VSV nAb. Considerably more BCR diversity is generated in VI10YEN mice than in T11µMT mice based on the following mechanisms: i) BCR editing at the knock-in locus [74], ii) usage of the endogenous IgH locus or of the endogenous IgL loci (incomplete allelic exclusion) and iii) affinity maturation. The latter as well as class switch recombination are possible at the knock-in locus which is of importance here since affinity maturation has been suggested necessary to generate LCMV nAbs [42].

Intact lymphoid structures are essential for priming of T- and B-cell responses [75,76]. Alerted by the known deficiencies of B cell-/- mice, we first validated our experimental models. We used a wide range of cell type-specific markers to perform an extensive comparative immunohistochemical analysis of the splenic microarchitecture in wild type C57BL/6, T11µMT, VI10YEN and B cell deficient µMT mice (Figure S1A and data not shown). T11µMT and VI10YEN mice exhibited a B220-positive B cell zone of normal size that was absent in µMT mice. The absence of B cells in µMT mice caused a compensatory enlargement of the red pulp as evident in increased numbers of F4/80-positive red pulp macrophages, alterations that were not seen in T11µMT and VI10YEN mice. As previously reported [34] µMT mice exhibited also a distorted marginal zone, reflected in a strong reduction or absence of ERTR9- and MOMA-1-positive marginal zone macrophages. Again, T11µMT and VI10YEN mice did not share these deficiencies, whereas analogous observations as in µMT mice were also made in B-cell-deficient JHT mice carrying a knock-out of the IgH joining region (not shown). Similarly also, 4C11-positive follicular dendritic cells were found in the spleen of T11µMT and VI10YEN mice, but were virtually absent from B cell-deficient animals (not shown).

Next we tested whether the normal lymphoid microarchitecture in VI10YEN and T11µMT mice supported unimpaired antiviral T cell responses. For this, µMT, T11µMT, VI10YEN and C57BL/6 wild type mice were infected with 10^6^ PFU of LCMV-WE intravenously (i.v). Eight days later we performed intracellular cytokine assays to measure CD4^+^ T cell responses against the viral GP64 and NP309 epitopes (Figure S1B-C), and CD8^+^ T cell responses against GP33 (Figure S1D). Day eight was chosen for analysis here since differences in viral load (compare Figures 4E-F) will over time inevitably result in impaired T cell responses [49,77,78]. Thus, potential differences in T cell responses later in the course of infection might have reflected a consequence of unchecked viral replication rather than the cause thereof. In accordance with previous reports [34] we found here that the CD8^+^ T cell response of µMT mice was normal whereas CD4^+^ T cell responses were impaired. In contrast, T11µMT and VI10YEN mice exhibited CD4^+^ and CD8^+^ T cell responses equivalent to C57BL/6 mice. These findings demonstrated that T11µMT and VI10YEN mice did not share the typical shortcomings of B cell-deficient mouse models and thus represented useful tools to address the need for specific antibody in preventing persistent viral infection. Of note, these observations matched a recent report on another B cell receptor transgenic mouse model with specificity for hen egg lysozyme [79].

Supporting references:

74. Casellas R, Shih TA, Kleinewietfeld M, Rakonjac J, Nemazee D, et al. (2001) Contribution of receptor editing to the antibody repertoire. Science 291: 1541-1544.

75. Muller S, Hunziker L, Enzler S, Buhler-Jungo M, Di Santo JP, et al. (2002) Role of an intact splenic microarchitecture in early lymphocytic choriomeningitis virus production. J Virol 76: 2375-2383.

76. Odermatt B, Eppler M, Leist TP, Hengartner H, Zinkernagel RM (1991) Virus-triggered acquired immunodeficiency by cytotoxic T-cell-dependent destruction of antigen-presenting cells and lymph follicle structure. Proc Natl Acad Sci U S A 88: 8252-8256.

77. Oxenius A, Zinkernagel RM, Hengartner H (1998) Comparison of activation versus induction of unresponsiveness of virus-specific CD4+ and CD8+ T cells upon acute versus persistent viral infection. Immunity 9: 449-457.

78. Wherry EJ, Blattman JN, Murali-Krishna K, van der Most R, Ahmed R (2003) Viral persistence alters CD8 T-cell immunodominance and tissue distribution and results in distinct stages of functional impairment. J Virol 77: 4911-4927.

79. McClellan KB, Gangappa S, Speck SH, Virgin HWt (2006) Antibody-independent control of gamma-herpesvirus latency via B cell induction of anti-viral T cell responses. PLoS Pathog 2: e58.
